# Analysis of the ways and methods of signaling pathways in regulating cell cycle of NIH3T3 at transcriptional level

**DOI:** 10.1186/s12860-015-0071-7

**Published:** 2015-10-28

**Authors:** Cuifang Chang, Zhipeng Niu, Ningning Gu, Weiming Zhao, Gaiping Wang, Yifeng Jia, Deming Li, Cunshuan Xu

**Affiliations:** College of Life Science, Henan Normal University, No. 46, Construction East Road, Xinxiang, 453007 Henan Province P. R. China; State Key Laboratory Cultivation Base for Cell Differentiation Regulation, Henan Normal University, Xinxiang, 453007 P. R. China

**Keywords:** NIH3T3 cell, Cell cycle, Signaling transduction, Gene expressional profile

## Abstract

**Background:**

To analyze the ways and methods of signaling pathways in regulating cell cycle progression of NIH3T3 at transcriptional level, we modeled cell cycle of NIH3T3 and found that G1 phase of NIH3T3 cell cycle was at 5–15 h after synchronization, S phase at 15–21 h, G2 phase at 21–22 h, M phase at 22–25 h.

**Results:**

Mouse Genome 430 2.0 microarray was used to detect the gene expression profiles of the model, and results showed remarkable changes in the expressions of 64 cell cycle genes and 960 genes associated with other physiological activity during the cell cycle of NIH3T3. For the next step, IPA software was used to analyze the physiological activities, cell cycle genes-associated signal transduction activities and their regulatory roles of these genes in cell cycle progression, and our results indicated that the reported genes were involved in 17 signaling pathways in the regulation of cell cycle progression. Newfound genes such as *PKC*, *RAS*, *PP2A*, *NGR* and *PI3K* etc. belong to the functional category of molecular mechanism of cancer, cyclins and cell cycle regulation HER-2 signaling in breast cancer signaling pathways. These newfound genes could promote DNA damage repairment and DNA replication progress, regulate the metabolism of protein, and maintain the cell cycle progression of NIH3T3 modulating the reported genes *CCND1* and *C*-*FOS*.

**Conclusion:**

All of the aforementioned signaling pathways interacted with the cell cycle network, indicating that NIH3T3 cell cycle was regulated by a number of signaling pathways.

**Electronic supplementary material:**

The online version of this article (doi:10.1186/s12860-015-0071-7) contains supplementary material, which is available to authorized users.

## Background

NIH3T3 is a mouse embryonic fibroblasts (MEFs) cell line with a high degree of contact inhibition in continued culture. It was separated from NIH Swiss mouse embryo cultures [1], and belongs to mesenchymal stem cells (MSCs)-like multipotent progenitor cells. These cellspossess multipotency and self renewal abilities [2], and are frequently used in transfection and gene expression researches [3]. Therefore, it is important to study the mechanism of signaling pathways that regulate NIH3T3 cell cycle, and to reveal more about cell cycle of NIH3T3 and the potential differentiation capability of MEFs. Furthermore, NIH3T3 also shows a promising prospect in clinical applications [1].

The self renewal of cells is a process of cell proliferation which includes nuclear division and cytokinesis. The process of nuclear division has four phases: G1 phase, S phase, G2 phase and M phase [4]. Dement et al. have analyzed cell cycle of NIH3T3 using flow cytometry after cells synchronized, and defined the times of the four phases (G1, S, G2 and M) of cell cycle [5]. Huang et al. found that the G1/S transition of NIH3T3 was affected when the Tet1 gene was knockout [6]. Study conducted by Lu et al. reported that over-expression of TIMP-1 gene promoted cell proliferation of NIH3T3 through p-AKT signaling pathway [7]. In addition, the work of Jeong et al. demonstrated that 2M4VP blocked the phosphorylation of Rb by regulating the proteins associated with cell cycle, and suppressed the cell growth of NIH3T3 that was treated with Bc-P [8]. According to previous researches,the cell cycle progression of NIH3T3 is regulated by a considerably high number of signaling pathways [9, 10].

In order to understand the regulatory mechanism of cell cycle-related signaling pathways in NIH3T3 cell cycle, we optimized the condition of synchronization and used Mouse Genome 430 2.0 microarray to detect the gene expression profiles of the NIH3T3 at ten different points in time. To our best knowledge, this is the first study to systematically analyze the whole signal transduction pathways of NIH3T3 cell cycle progression.

## Results

### The modeling of the NIH3T3 cell cycle

**Fig. 1:**
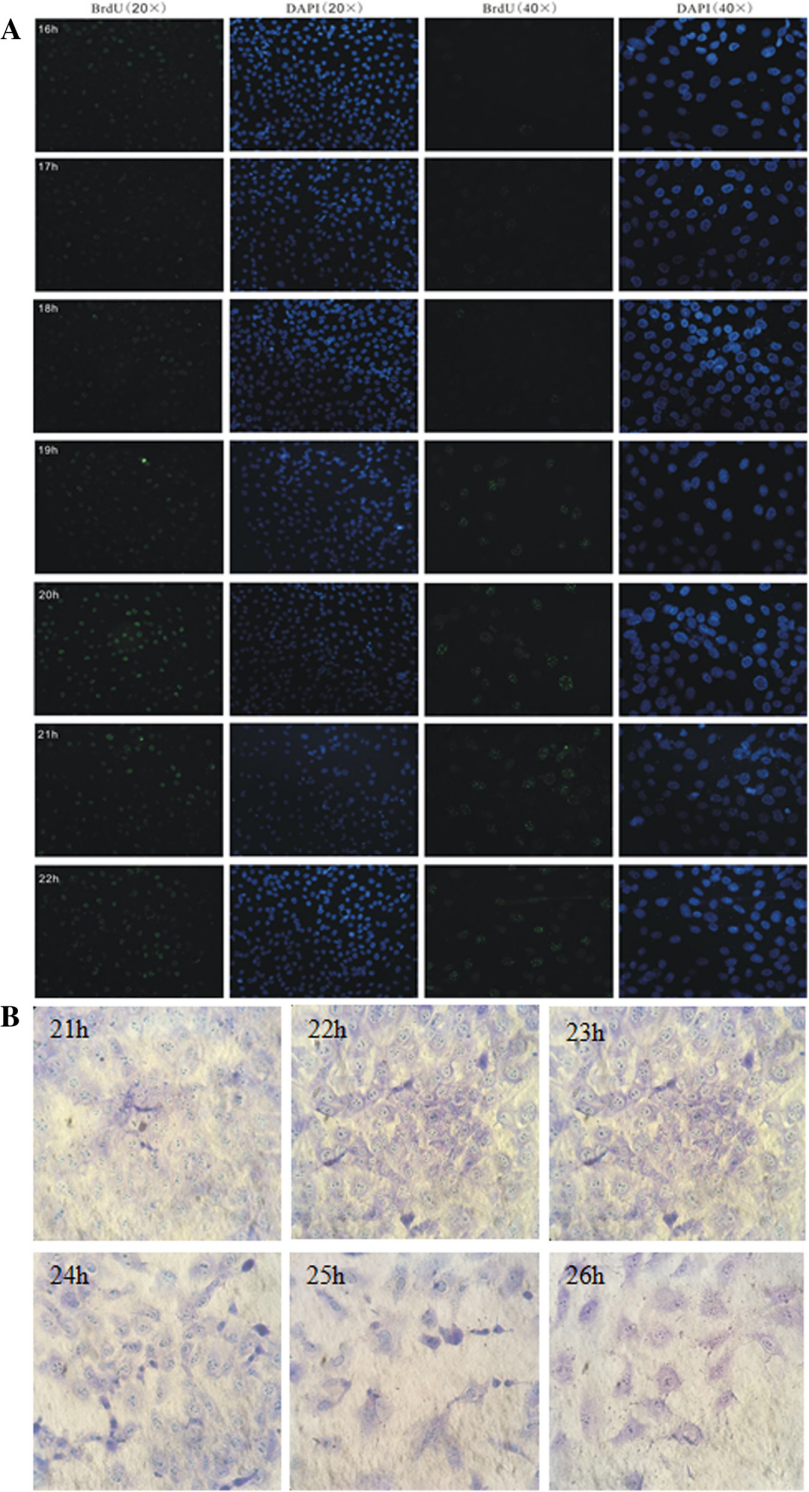
Build of NIH3T3 cell cycle **a**. S phase cell detected by immunocytochemistry at different time points after synchronization; **b**. M phase cell detected by morphologic observation at different time points after synchronization

The S phase and the M phase of NIH3T3 cell cycle were detected by immunocytochemistry and morphologic observation. The results of immunocytochemistry showed that the ratios of S phase-positive of cells collected at 16, 17, 18, 19, 20, 21, 22 and 23 h after synchronization were 1.94 %, 9.03 %, 17.27 %, 35.74 %, 53.56 %, 72.41 % and 88.12 %, respectively (Fig. 1a), indicating that the S phase was at 15 ~ 21 h after synchronization. Morphologic observation revealed that the ratios of the cells in mitosis phase collected at 21, 22, 23, 24, 25 and 26 h were 1 %, 2 % 20 %, 40 %, 40 %, 25 %, respectively (Fig. 1b), indicating that M phase was at 22 ~ 25 h after synchronization. The results also showed that when the cells were synchronized by the method mentioned above, the G1 phase of the cells could last for 5 h. In summary, we found that G0/G1 phase checkpoint of NIH3T3 cells was at 5 h after synchronization, G1 phase at 5–15 h, G1/S phase checkpoint at 15 h, S phase at 15–21 h, S phase checkpoint at 21 h, G2 phase at 21–22 h, G2/M phase checkpoint at 22 h, M phase at 22–25 h, end of M phase at 25 h.

### Expression profiles of genes associated with NIH3T3 cell cycle

Expression profiles of the genes associated with cell cycle of NIH3T3 were detected by Mouse Genome 430 2.0 microarray. 1024 genes were found to be associated with cell cycle of NIH3T3, of which 595 genes were up-regulated, 417 genes were down-regulated, and 12 genes were up/down-regulated (Table 1, Additional file 1: Table S1). Furthermore, only 64 of the 1024 genes have been reported, while the remaining 960 genes were newfound genes related to cell cycle. The ratio values of the cell cycle-associated genes were uploaded to “Dataset Files” of IPA software to analyze the physiological activity in which the significant expressed genes were involved. The physiological activity coefficient –log (*p*-value) was calculated by “Comparison Analyses”. Ours results revealed that these genes were involved in a remarkably high number of physiological activities, including cell development, cell death and survival, cell growth and proliferation, cell cycle, DNA replication, recombination and repair, etc. (Additional file 2: Figure S1).

**Table 1:** Expression changes of genes-related cell cycle in NIH3T3 cells

Genes	Time of re-enter into cell cycles after synchronization (h)																		Sum
	5		10		15		18		21		21.5		22		23.5		25		
	Reported	Newfound	Reported	Newfound	Reported	newfound	Reported	Newfound	Reported	Newfound	Reported	Newfound	Reported	Newfound	Reported	Newfound	Reported	Newfound	
Up-regulated	5	262	13	235	10	317	17	316	4	323	10	314	6	306	1	307	14	303	595
Down regulated	3	153	8	182	2	168	4	172	-	161	3	130	2	142	1	134	1	139	417
Up/Down-regulated	-		-		-		-		-		-		-		-		-		12
Sum	423		438		497		509		488		457		456		443		457		1024

7

### Reliability of the microarray check results

**Fig. 2:**
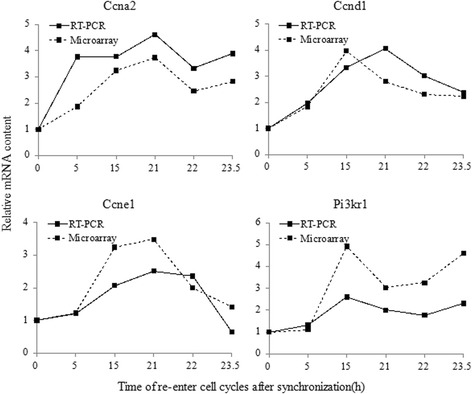
mRNA expression of four selected genes measured by microarrays and RT-PCR. Solid line presented the results of RT-PCR and dotted line that of Rat Genome 230 2.0 Array

To validate the reliability of microarray check results, qRT-PCR was used to detect the expression changes of *CCNA2*, *CCND1*, *CCNE1* and *PIK3R1* in NIH3T3 cell cycle. The results showed that qRT-PCR detected gene expression pattern similar to pattern detected by microarray (Fig. 2).

**Fig. 3:**
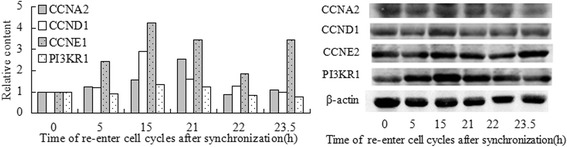
Expression level of four selected proteins measured by Western blot

In order to further confirm the correlation of gene expression changes and protein expression, we used Western blot analysis to examine the expression changes of six proteins, CCNA2, CCND1, CCNE1 and PIK3R1. The results showed a significant up-regulation in the expression of CCNA2 and CCNE1 at 15 h and 21h, CCNB1 at 23.5 h, CCND1 at 15 h, PIK3R1 at 15–23.5 h, and reduction in the expression of FOS at 5–23.5 h (Fig. 3), suggesting that the protein expression pattern detected by Western blot was similar to gene expression pattern detected by microarray and qRT-PCR.

### The physiological activities and signal transduction activities in which cell cycle associated genes involved

**Fig. 4:**
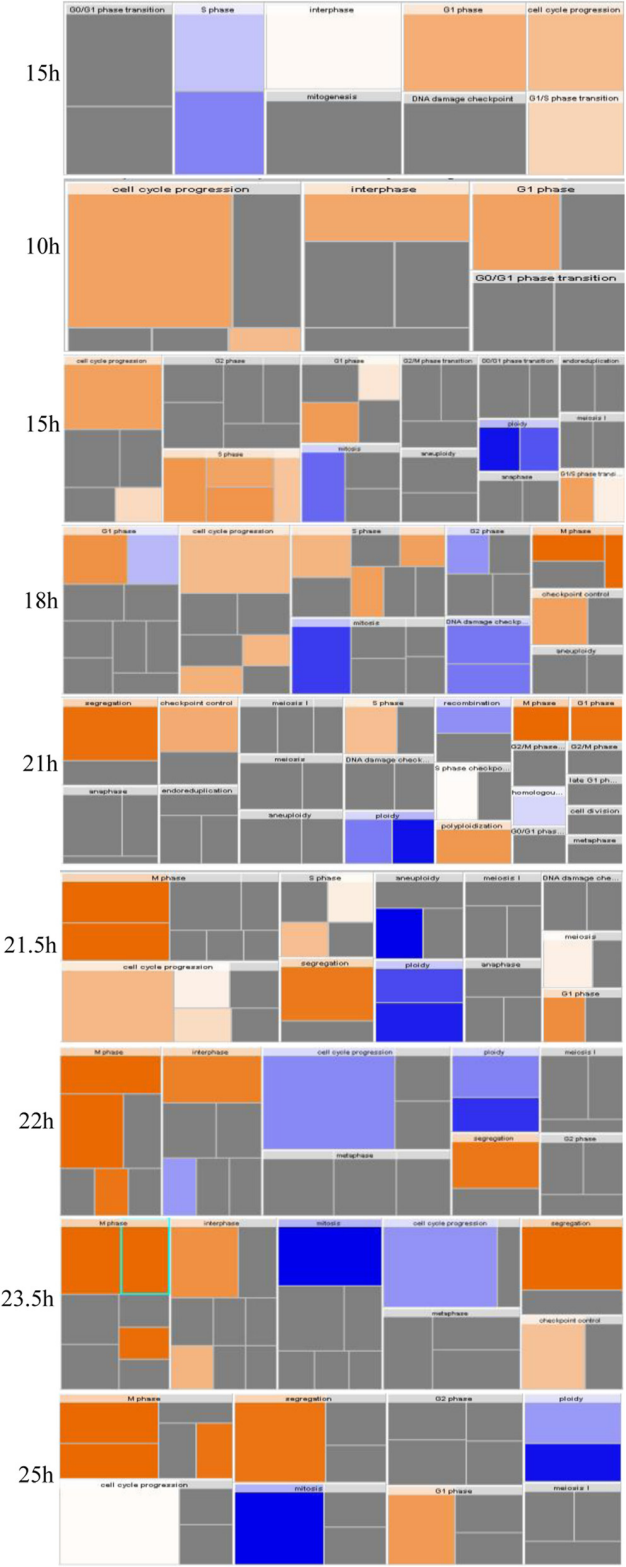
The Genes heat maps of physiological activity the genes involved at different time of cell cycle

The analysis of the cell cycle physiological activities, which involved the reported cell cycle genes at different points in time, demonstrated that “G1 phase” and “cell cycle progression” were stronger at 5 h after synchronization, “G1 phase” and “cell cycle progression” at 10 h, “G1/S transition” at 15 h, “S phase” and “cell cycle progression” at 18 h, “M phase” and “checkpoint” at 21 h, S phase, “M phase” and “cell cycle progression” at 21.5 h, “M phase” at 22 and 23.5 h, “M phase” and “separation” at 25 h. Overall, the physiological activities conformed with cell cycle progression at all these points in time (Fig. 4).

Following the previous analysis, the coefficients–log (*p*-value) of the signaling pathways in which the genes involved were detected, and analysis discovered that the coefficients–log (*p*-value) of the signaling pathways in which the genes involved were detected, and the analysis discovered that that 17 signaling pathways could contribute to the modulation of cell cycle, including 14–3–3 mediated signaling, antiproliferative role of somatostain receptor 2, aryl hydrocarbon receptor signaling, CDK5 signaling, ceramide signaling, DNA damage-induced 14–3–3σ signaling, G2/M DNA damage check point regulation, integrin signaling, role of CHK proteins in cell cycle checkpoint control and tight junction signaling etc (Table 2) (Additional file 3: Figure S2).

**Table 2:** Signaling pathways that cell cycle-associated genes involved at different time points

Time	5h	10h	15h	18h	21h	21.5h	22h	23.5h	25h
Cell cycle	G0/G1 check point	G1 phase	G1/S check point	S phase	S check point	G2 phase	G2/M check point	M check point	End of M phase
14-3-3-mediated Signaling	~	-	+/-	~	-	~	~	~	~
Antiproliferative Role of Somatostatin Receptor 2	-	+	-	+	~	~	~	~	+
Aryl Hydrocarbon Receptor Signaling	+/-	+/-	+/-	+/-	+/-	+/-	+/-	+/-	+/-
ATM Signaling	+/-	-	+/-	+/-	+/-	+/-	+/-	-	-
CDK5 Signaling	+/-	-	-	+/-	+/-	+/-	+/-	+/-	+/-
Cell Cycle Control of Chromosomal Replication	~	+	+	+	+	+	+	+	+
Ceramide Signaling	-	+	+	+	+	+	+	~	+
Cyclins and Cell Cycle Regulation	+/-	+	+	+	+	+	-	-	-
DNA damage-induced 14-3-3σ Signaling	-	+/-	+/-	+/-	+/-	+/-	+	+	+
Estrogen-mediated S-phase Entry	+	+	+	+	+	+	+	+	+
G1/S Check Point Regulation	~	+	+	+	+	+	+	+	+
G2/M DNA Damage Check Point Regulation	+	+	~	+	+	+	+	+	+
GADD45 Signaling	+/-	+	+/-	+/-	+/-	+/-	+	+	+
Integrin Signaling	+/-	+/-	+/-	+/-	+/-	~	~	~	~
Mitotic Role of Polo-Like Kinase	+	+	+	+	+	+	+	+	+
Role of CHK Proteins in Cell Cycle Checkpoint	-	-	-	-	-	-	-	-	-
Tight Junction Signaling	-	-	-	+	~	~	~	~	-

^a^ “+” represents the signaling pathway promote the down-stream physiological activity associated with cell cycle progression, “-” represents the signaling pathway inhibit the physiological activity, “~” represents the signaling pathway has little impact on the physiological activity

### Signal transduction activities of the newfound cell cycle associated genes

**Fig. 5:**
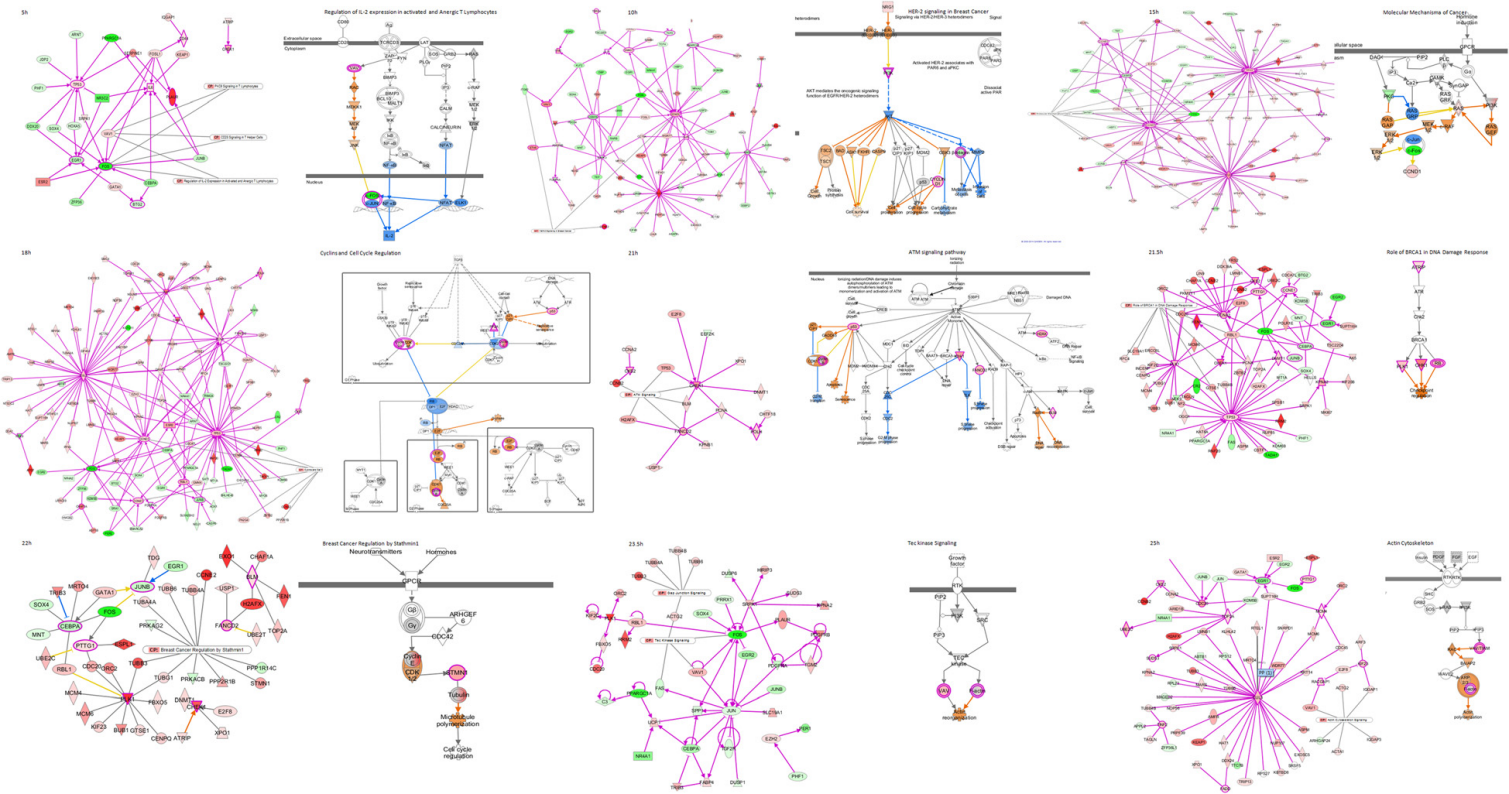
Interaction between newfound and reported genes associated with cell cycle. Symbols in purple box present the genes have been reported to be associated with cell cycle, symbol under red ground the up-regulate genes, those under green the down-regulate

The analysis of the newfound genes associated with cell cycle showed that newfound genes could regulate reported genes *CCND1* and *C*-*FOS* etc. through signaling pathways of “molecular mechanisms of cancer”, “cyclins and cell cycle regulation”, “HER-2 signaling in breast cancer” etc., and promote DNA repair, DNA replication, protein metabolism and cell cycle progression (Fig. 5).

### The interaction between the cell cycle-associated signaling pathways and cell cycle gene network

**Fig. 6:**
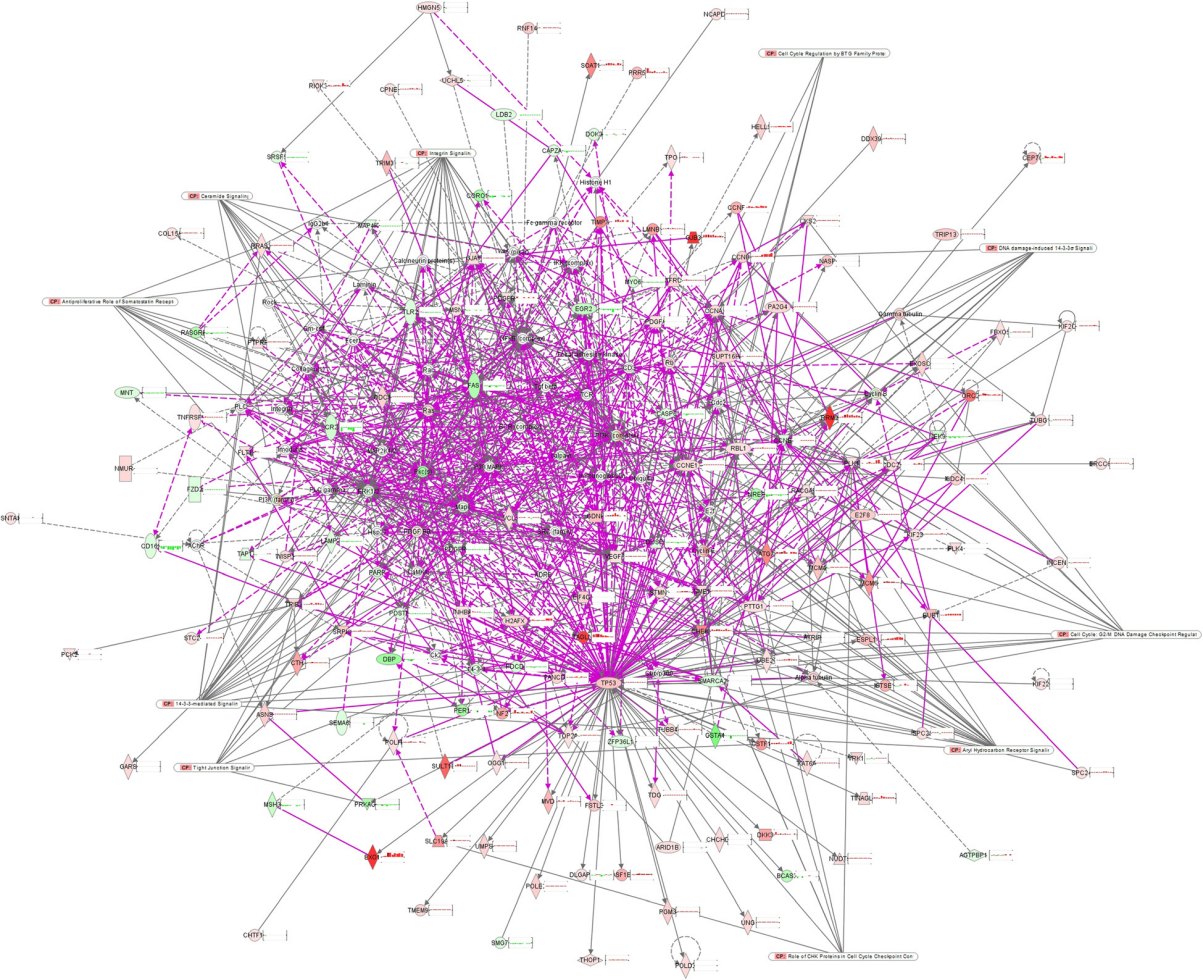
The interaction between the cell cycle-associated signaling pathways and cell cycle network purple box present the genes have been reported to be associated with cell cycle, symbol under red ground the up-regulate genes, those under green the down-regulate

IPA was used to analyze the interaction between the cell cycle-associated signaling pathways and cell cycle gene network at different time points. The results showed that different signaling pathways were involved in the regulation of cell cycle progression at different time points (Additional file 4: Figure S3), but all of them were involved in the regulation of cell cycle progression (Fig. 6). Further analysis of the upstream regulators which may play a predominant role revealed that, at the gene transcription level, *FOS*, *JUN*, *FOSL1* and *EGR1* began to contribute at 5 h after synchronization; *TP53*, *JUN* and *FOSL1* at 10 h; *TP53*, *CCND1* and *FOSL1* at 15 h; *TP53*, *CCND1* and *TRIM24* at 18 h; *TP53* and *TOB1* at 21 h; *TP53* and *CCND1* at 21.5 h; *TP53* at 22 h; *TP53* and *KDM5B* at 23.5 h; *TP53* and *KDM5B* at 25 h.

## Discussion

MEFs have attracted an increasing amount of attention for its potential role in expounding stem cell differentiation and its application in analyzing the gene expression. NIH3T3 is a MEFs cell line isolated from NIH Swiss mouse embryo cultures, and the study of its cell cycle has important biological science significance. Using IPA, we researched the expression profiles of the cell cycle-associated genes, signaling pathways associated with cell cycle and signal transduction activities of cell cycle-associate signaling pathways at different time points, and the results showed that signaling pathways of “molecular mechanism of cancer” and “HER-2 signaling in breast cancer” etc. were associated with cell cycle progression, but played different roles at different time points.

Previous studies proved that proto-oncogene c-Fos had an important role in G0/G1 transition, and inhibited cell proliferation in some cell types [11]. In the regulation of interleukin 2 (IL-2) expression in activated and anergic T lymphocytes signaling pathway, T cell receptor (TCR) was activated by antigen, which activated the transcription factor AP1 through TCR → VAV → RAC → JNK → AP1, and AP1 was a heterodimer formed by c-Fos and c-Jun and promoted apoptosis via downstream molecules IL-2 [12–14]. In this study, *VAV* was up-regulated at 5 h, *C*-*FOS* was down-regulated at 5 h, and further analysis of the physiological activities predicted by expression profiles of signaling pathway-associated genes by IPA indicated that the activity of IL-2 related to apoptosis was inhibited. It was speculated that the aforementioned signaling pathway could promote G0/G1 transition of NIH3T3.

Wu et al. demonstrated that, v-erb-b2 avian erythroblastic leukemia viral oncogene homolog 3 (ErbB3) could promote cell proliferation of tumor cells through PI3K/AKT after it was activated [15]. AKT, when activated, could phosphorylate and inhibit GSK3, which in turn activate CCND1, and promote cell proliferation and cell cycle progression [16]. Germain et al. indicated that CDK2-CyclinE complexes promoted ubiquitination of proteins [17]. Similarly, our study here reveals that at 10 h, *NGR1* which act as the ligands of *ERBB3* was up-regulate , *PI3K* was down-regulate, *CCND1* was up-regulate, and further analysis revealed that cell proliferation and cell cycle progression were promoted by the signaling pathways. Therefore, we conclude that CCND1, which belongs to the components of CDK2 and the downstream molecules of HER-2 signaling in Breast Cancer signaling pathway, could promote G1 phase of NIH3T3 by participating in the synthesis and degradation of protein.

G protein-coupled receptors (GPCR) could activate small G protein (RAS) via PLCβ, DAG and PKC in turn, and via PI3Ks. Then, the downstream molecules c-RAF, MEK1/2, and ERK1/2 were activated in turn. RAS could also activate CCND1 via AP1 [18, 19]. Parrales et al. pointed out that CCND1 which was activated by ERK via c-FOS could promote G1/S transition [20]. The results of this study showed that at 15 h, *PKC*, *RAS* and *CCND1* were up-regulated, *c*-*FOS* was down-regulated, and further analysis demonstrated that the CCND1 which related to cell cycle was activated. In summary, we conclude that molecular mechanism of cancer signaling pathway could promote G1/S transition of NIH3T3, but further study is needed to understand the mechanism.

Previous studies found that protein phosphatase 2A (PP2A) could promote the phosphorylation of Rb via inhibiting the activity of CDK2-CyclinE complex [21, 22], which enables E2F and promotes DNA replication to be released from Rb-E2F complex [23]. In this study, *PP2A* and *CyclinE* were up-regulate at 18 h, and further analysis showed that S phase was promoted. It is concluded that cyclins and cell cycle regulation signaling pathway could promote S phase of NIH3T3.

Ataxia-telangiectasia mutated (ATM) could inhibit TLK via phosphorylating CHK1, and arrest S phase [24]. ATM also could phosphorylate H2AX [25], and promote DNA repair via activating ATF2 [26]. It has been reported that ATM signaling pathway played a key role in the regulation of cell cycle checkpoints when the DNA was damaged [27]. In this study, *CHK1* and *H2AX* were both up-regulated at 21 h, and further analysis declared that DNA repair was promoted. Hence, we hypothesise that the above mentioned signaling pathway could promote DNA repair when the DNA was damaged, and promote NIH3T3 to pass the S phase checkpoint.

As recently reported, GPCR could activate G proteins when combined with ligand, and activate CDC42 via interacting with ARHGEF6 [28, 29]. Then STMN1 was phosphorylated by the activated CDK2-CyclinE complex [30, 31], and could promote tubulin polymerization [32, 33]. Our study here reveals that *STMN1*, *CyclinE* family members *ACTG2* and *CCNE2*, and *Tubulin* family members *TUBG1*, *TUBB4A*, *TUBB4B*, *TUBB6H2AX* were all up-regulated at 22 h, and further analysis manifested that Tubulin polymerization was promoted. In summary, we speculate that the signaling pathway mentioned above could prepare for the entry of M phase of NIH3T3 via promoting Tubulin polymerization at G2 phase.

Previous studies indicated that ATR could activate BRCA1 via CHK2 when it interacts with ATRIP [34, 35], and promote G2/M phase transition via PLK1 [36, 37], and BRCA1 also could? inhibit G2/M transition via activating CHK1 or interacting with Rb [38]. Jalili et al. pointed out that PLK1 promoted G2/M transition, and the expression of PLK1 peaked at G2/M transition [39]. In this study, *ATRIP*, *CHK1*, *PLK1* and *RB* were all up-regulated at 22h, and further analysis showed that the regulation of G2/M transition was enhanced. It is speculated that BRCA1 in DNA damage response signaling pathway promote G2/M transition.

It was demonstrated that RTK could promote PIP2 turn to PIP3 via PI3K when it was activated by ligands [40], and then PIP3 could activate the downstream molecule TEC, while TEC also could be activated by RTK via SRC and then promote actin reorganization via VAV and F-actin [41]. The research of Laird et al. proved that connexin could promote reorganization of cytoskeleton via interacting with F-actin when it interacts with DBN1 [42]. Lancaster et al. revealed that the reorganization of cytoskeleton was very important for M phase [43]. In this study, *VAV* family member *VAV1* and *F*-*actin* family member *ACTG2* were both up-regulated at 23.5 h, and further analysis provided evidence that the reorganization of actin and cytoskeleton were promoted. Therefore, we imply that Gap junction and Tec kinase Signaling pathways could promote M phase of NIH3T3.

RTK could lead to the activation of its downstream molecules SHC, GRB2, RAS, PI3K in turn [44], and promote to activate PIP2 into PIP3 [45], and then PIP3 activate APR2/3-F-actin via activating VAV/TIAM [46], RAC, BAIAP2 and WAVE2 in turn, and promote synthesis of actin [47]. In this study, *VAV* family member *VAV1* and *F*-*actin* family members *ACTA1* and *ACTG2* were all up-regulated at 25 h, and further analysis of the physiological activities predicted by expression profiles of signaling pathway-associated genes indicated that the synthesis of actin was promoted. It is speculated that Actin Cytoskeleton Signaling pathway could promote M phase of NIH3T3 by promoting the formation of contractile ring.

In summary, we found that *VAV* and *c*-*FOS* play a key role at 5 h, *NGR1* and *CCND1* at 10 h, *c*-*FOS* and *CCND1* at 15 h, *PP2A* and *CyclinE* at 18 h, *CHK1* and *H2AX* at 21 h, *STMNA* and *CyclinE* at 21.5 h, *ATREP* and *PLK1* at 22 h, *F*-*actin* and *VAV* at 23.5 h, *F*-*actin* and *VAV* at 25 h. These genes promoted various physiological activities to proceed methodically by the interactions of different signaling pathways, and then promoted the progression of NIH3T3 cell cycle. The results will be validated in our future studies by using gene over-expression, gene knockout, RNA interference, inhibitors of signaling pathways, and so on.

## Conclusion

*VAV* and *c*-*FOS* play a key role at 5 h of NIH3T3 cell cycle, *NGR1* and *CCND1* at 10 h, *c*-*FOS* and *CCND1* at 15 h, *PP2A* and *CyclinE* at 18 h, *CHK1* and *H2AX* at 21 h, *STMNA* and *CyclinE* at 21.5 h, *ATREP* and *PLK1* at 22 h, *F*-*actin* and *VAV* at 23.5 h, *F*-*actin* and *VAV* at 25 h.

## Methods

### Preparation and identification of cell cycle model of NIH3T3

Dement et al. analyzed the cell cycle of NIH3T3 with synchronized cells. They also studied cell populations at different time points by immunofluorescence approach, and determined the times of the four phases (G1, S, G2 and M) of cell cycle [5]. Based on their study, we explored the best condition for synchronization. The total cells count of 8 × 10^4^ (1 × 10^4^ /ml medium) were inoculated into the medium with 10 % serum and cultured for 3 days at 37 °C (cell density would reach to 2 × 10^4^ /cm^2^), and the medium was then replaced with 5 % serum and the cells were cultured for 2 days at 37 °C to allow cells in different phases of cell cycle to enter G0 phase [48], then transferred into the medium with 10 % serum and cultured at 37 °C. After that, the cells were collected at 16, 17, 18, 19, 20, 21, 22, 23, 24, 25 and 26 h. Through morphology observation and BrdU immunocytochemistry detection, the times of different phases of cell cycle of NIH3T3 were determined. The cells were collected for further research at each of the following time points: 5, 10, 15, 18, 21, 21.5, 22, 23.5 and 25 h after synchronization, meanwhile the cells in the control group were collected at 0 h [49].

### Mouse Genome 430 2.0 microarray detection and data analysis

Total RNA of NIH3T3 cells was extracted and purified according to the protocol as previously described [50]. The first cDNA chain was synthesized by SuperScript II RT reverse transcription system, and the second chain was synthesized following the guideline of Affymetrix cDNA kit. Biotin-labeled cRNA was prepared using GeneChip In Vitro Transcript Labeling Kit (ENZO Biochemical, New York, NY) as instructed by the manufacturer. cRNA fragments of 35–200 bp were obtained by fragmentation reagent treatment [51]. Then, the prehybridized Mouse Genome 430 2.0 Array was hybridized with the cRNA fragments that were pretreated. Subsequently, they were washed and stained automatically using GeneChip® Fluidics Station 450 (Affymetrix Inc., Santa Clara, CA, USA), scanned and imaged with a GeneChip scanner 3000 (Affymetrix Inc., Santa Clara, CA, USA). The images reflecting gene expression abundance were converted into normalized signal values using Affymetrix GCOS 2.0 software [52]. The genes were defined as present (*P* < 0.05), marginal (0.05 < *P* < 0.065) and absent (*P* > 0.065) according to the *P*-values of the probe signal. Then, the data of each array was initially normalized, and the ratio at each time point was calculated through the normalized signal values of experimental group compared to control group (0 h). To minimize the experimental operation and microarray test errors, each sample was repeated three times, and the average value was used in subsequent statistical analysis.

### Quantitative real-time polymerase chain reaction (qRT-PCR)

To validate the reliability of Mouse Genome 430 2.0 array, the expression level of genes *CCNA2*, *CCND1*, *CCNE1* and *PIK3R1* were detected by qRT-PCR in NIH3T3 cell cycle progression. Primers for these genes were designed using Primer Express 5.0 software. The first chain of cDNA was synthesized by SuperScript II RT reverse transcription system (Promega, USA). The PCR were performed by the conditions with SYBR Green I: 2min at 95 °C, followed with 40 cycles for 15s at 95 °C, 15s at 60 °C, and 30s at 72 °C. Each sample was performed in triplicates. β-actin was used as internal reference.

### Western blot analysis

Western blot analysis was performed according to Towbin’s method [16]. 100μg proteins were separated by SDS-PAGE and then transferred to a PVDF membrane (GE Healthcare). After that, the membranes were blocked with 5 % skimmed milk in Tris-buffered saline (TBS) containing 0.1 % Tween-20 (TBS-T), and subsequently incubated overnight at 4 °C with the respective primary antibodies rabbit anti-CCNA2, -CCND1, -CCNE1 and -PIK3R1 (Bioss, 1:1,000). After washing with TBS-T for 30 minutes at room temperature, the membrane was further incubated with horseradish peroxidase-conjugated secondary antibodies goat anti-rabbit IgG (Sigma, 1:5,000) for 1.5 h at 37 °C. Finally, protein bands were visualized with Amersham ECL substrates. The relative abundances of target proteins was measured by Image analysis. β-actin (sigma, 1:1,000) was served as internal reference.

### Identification of cell cycle associated genes of NIH3T3

The genes with ratio values ≥3 or ≤0.33 were regarded as significant expressed genes. Specifically, the genes with ratio values ≥3 were considered as up-regulated genes, ≤0.33 as down-regulated genes, and between 0.33–3 as insignificantly changed genes.

### Identification of genes and signaling pathways associated with cell cycle

The cell cycle-associated genes were identified by three methods. First “cell cycle” was used as the keyword to search for all the genes were associated with cell cycle that have been deposed in Gene Ontology (www.geneontology.org), NCBI (www.ncbi.nlm.nih.gov/.nlm.nih.gov) and RGD (rgd.mcw.edu). Second, the genes-associated with cell cycle were confirmed by the Ingenuity Pathway Analysis 9.0 software (IPA), KEGG (www.genome.jp/kegg/pathway.html) and QIAGEN (www.qiagen.com/geneglobe/pathways.aspx) [53, 54]. Finally, the list of cell cycle associated genes was further extended and supplemented by consulting relevant literature. In conclusion, the above-described genes were integrated and regarded as cell cycle associated genes.

The cell cycle-associated signaling pathways were computed through two approaches. One was to enter the key word “cell cycle” in the websites of QIAGEN and KEGG to obtain cell cycle-associated signaling pathways. The other was to upload the genes associated with cell cycle into the “Canonical Pathway” frame of the IPA software to obtain cell cycle-associated signaling pathways [55]. Finally, the signaling pathways that appeared in both methods were selected and regarded as cell cycle associated pathways.

### Identification of newfound genes and signaling pathways associated with cell cycle

The newfound genes were obtained by comparative analysis of NIH3T3 cell cycle-associated genes detected by microarray and the reported cell cycle-associated genes. After uploading the reported genes and the newfound genes to the “Build → Path explorer” frame of the IPA software, the interaction network between the genes were establish, and the signaling pathways which they involved were looked through the “Overlay → Canonical Pathway” frame. Signaling pathways which the reported genes and the newfound genes both involved, and which only the newfound genes involved but involved in cell proliferation and cell cycle physiological activities were considered as the newfound signaling pathways associated to cell cycle in which the newfound cell cycle-associated genes involved. The signaling pathways with newfound genes involved were considered as newfound signaling pathways.

### Interaction of signaling pathways and cell cycle networks

The interaction networks with the genes associated with cell cycle of NIH3T3 at different time points of cell cycle were analyzed using IPA, and the upstream regulatory factors which may play a key role and theirroles were also predicted and analyzed using IPA.

## Additional files

Additional file 1: Table S1. Expression profiles of cell cycle-associated genes. (XLSX 163 kb) Additional file 2: Figure S1. Physiological activity that the reported genes involved. (DOC 580 kb) Additional file 3: Figure S2. Signal transduction of the signaling pathway that the reported genes involved (DOC 4343 kb) Additional file 4: Figure S3. The interaction network between signaling pathway and cell cycle network. (DOC 9794 kb)

## Abbreviations

MEFs: Mouse embryonic fibroblasts
MSCs: Mesenchymal stem cells
