# Supplementary figures and images for: Analysis of the ways and methods of signaling pathways in regulating cell cycle of NIH3T3 at transcriptional level
Source: BMC Cell Biol. 2015 Oct 28;16:25. doi: 10.1186/s12860-015-0071-7 (PMC4625951; doi:10.1186/s12860-015-0071-7)

Additional file 2: Figure S1 Physiological activity that the reported genes involved


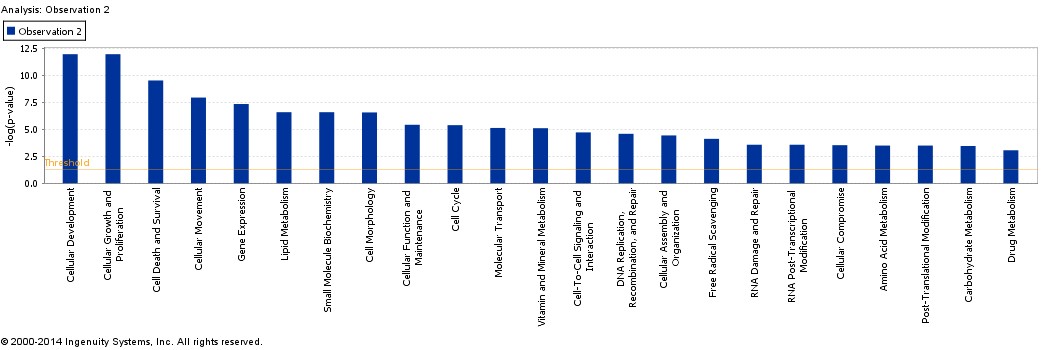

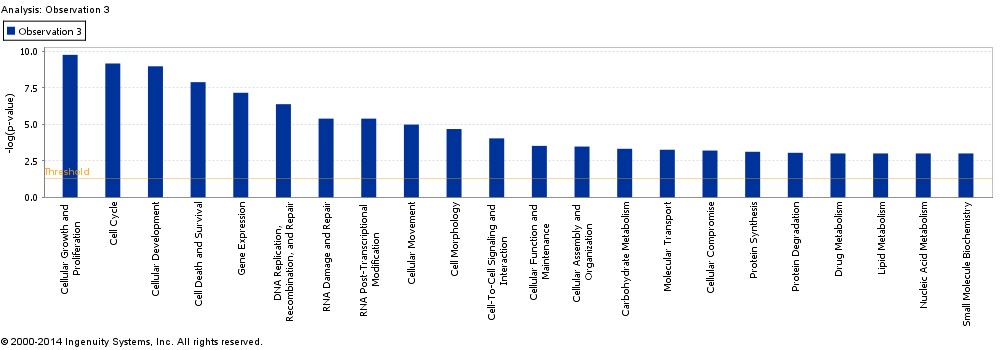

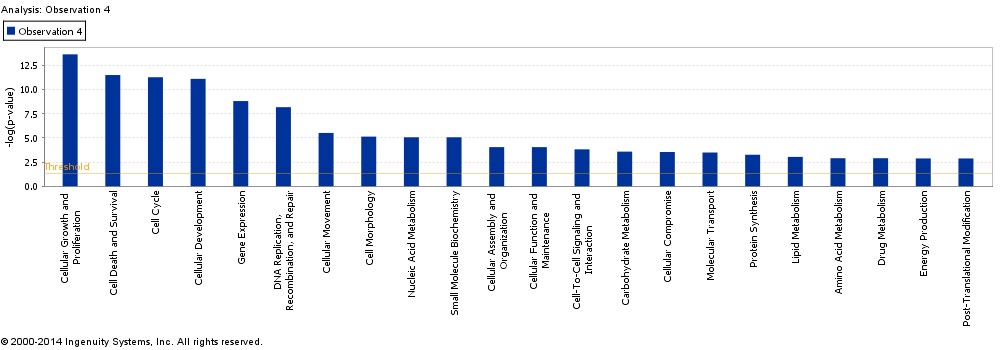

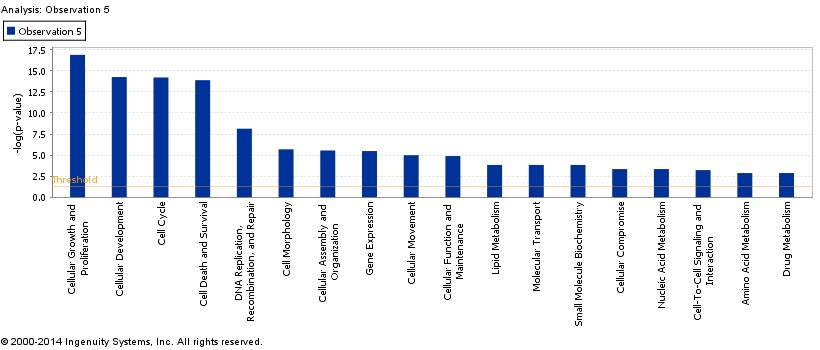

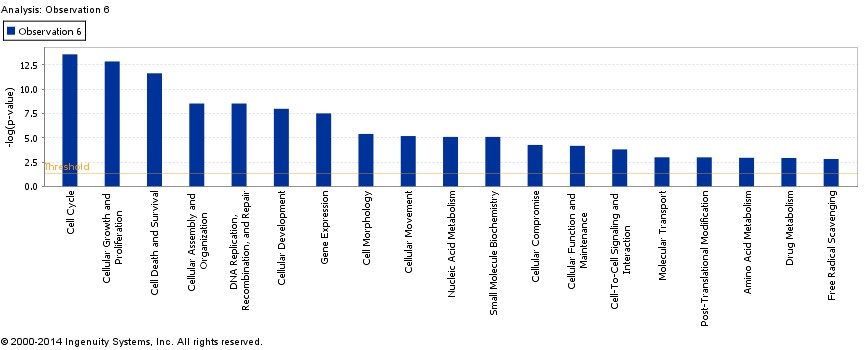

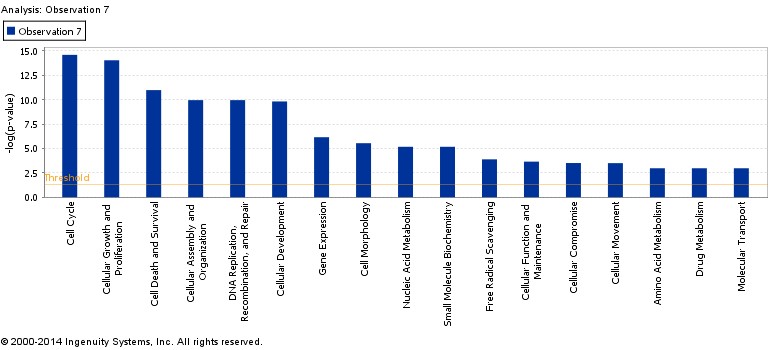

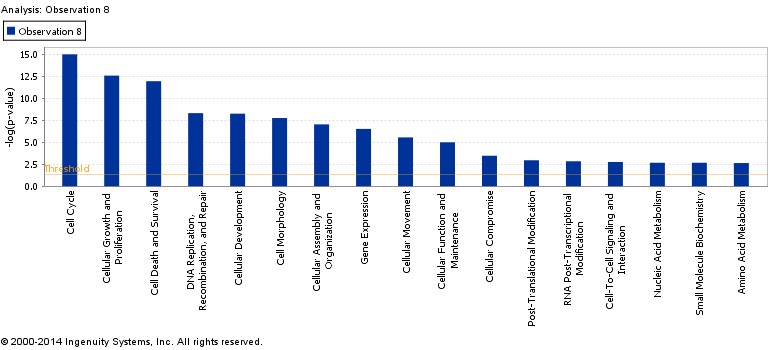

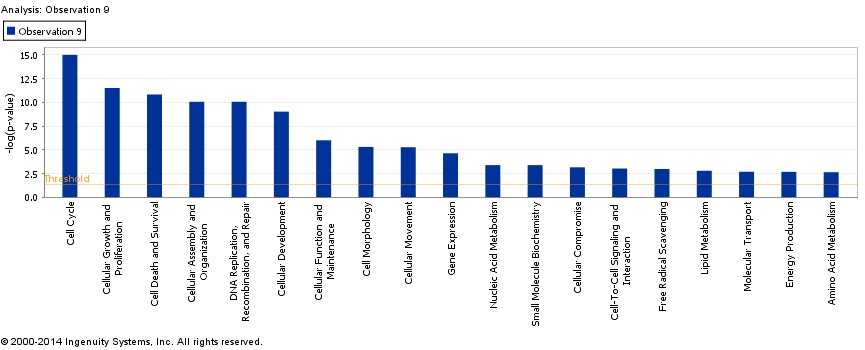

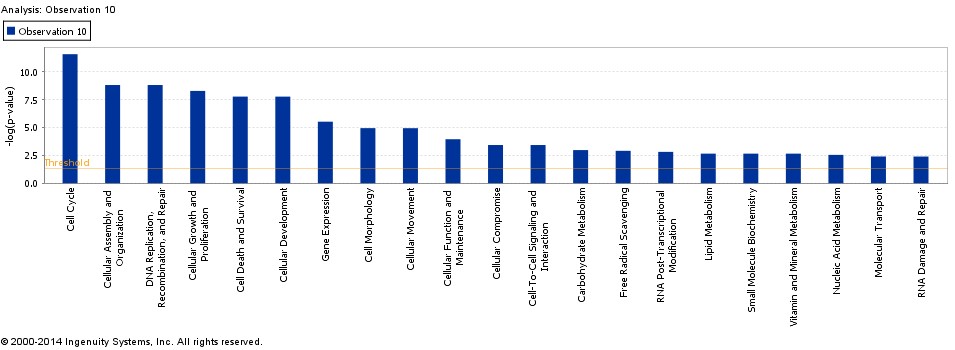


10h

15h

18h

21h

21.5h

22h

23.5h

25h

5h

Supplement: Additional file 2: Figure S1. — Physiological activity that the reported genes involved. (DOC 580 kb) [file 12860_2015_71_MOESM2_ESM.doc]

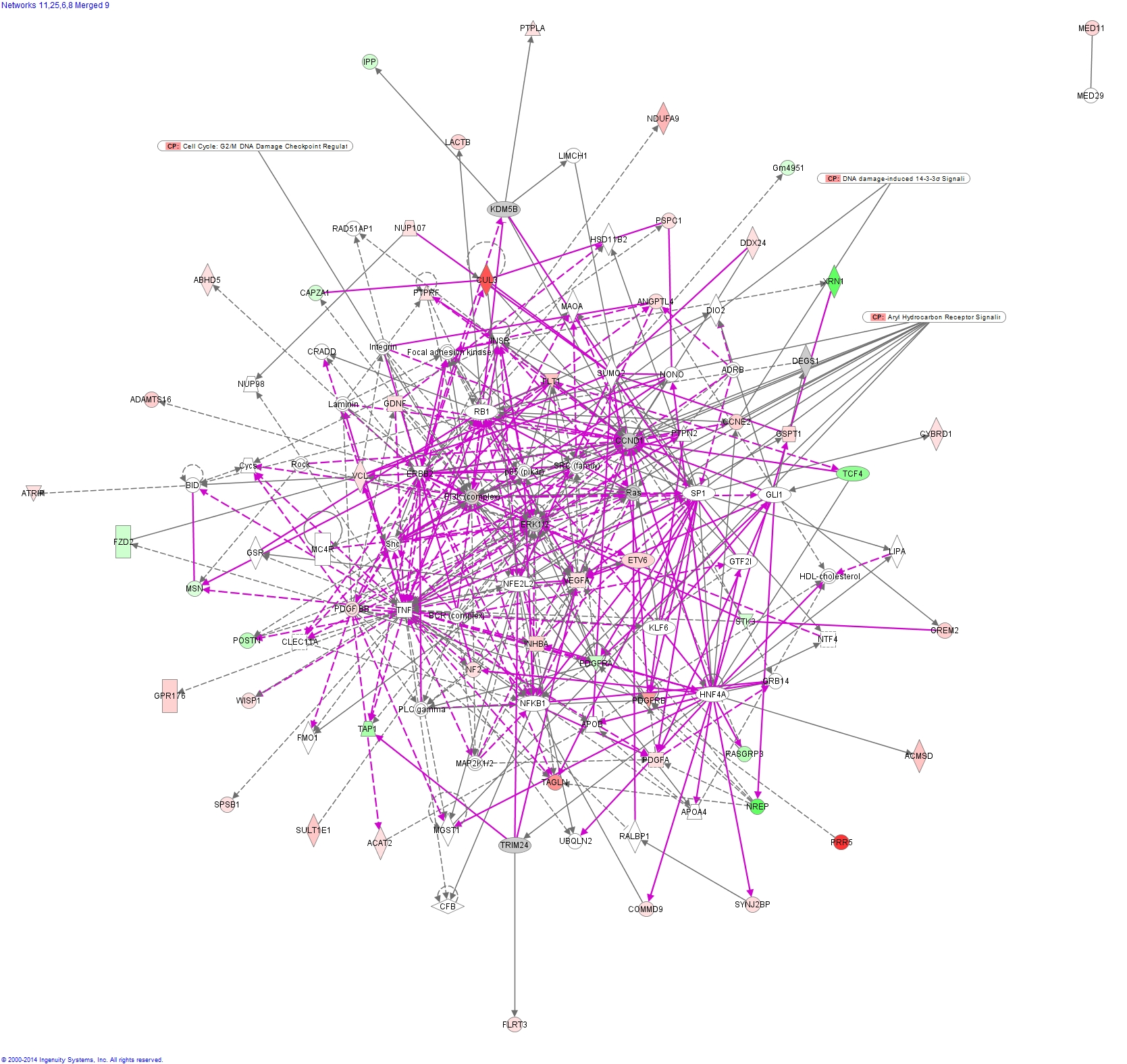


5h

10h


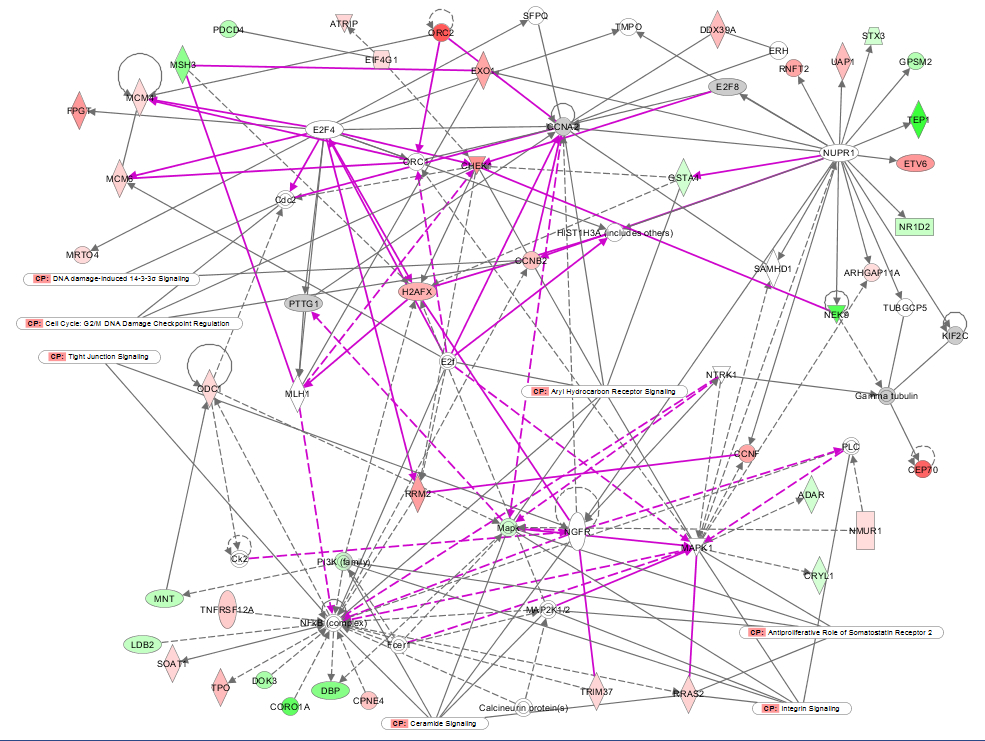


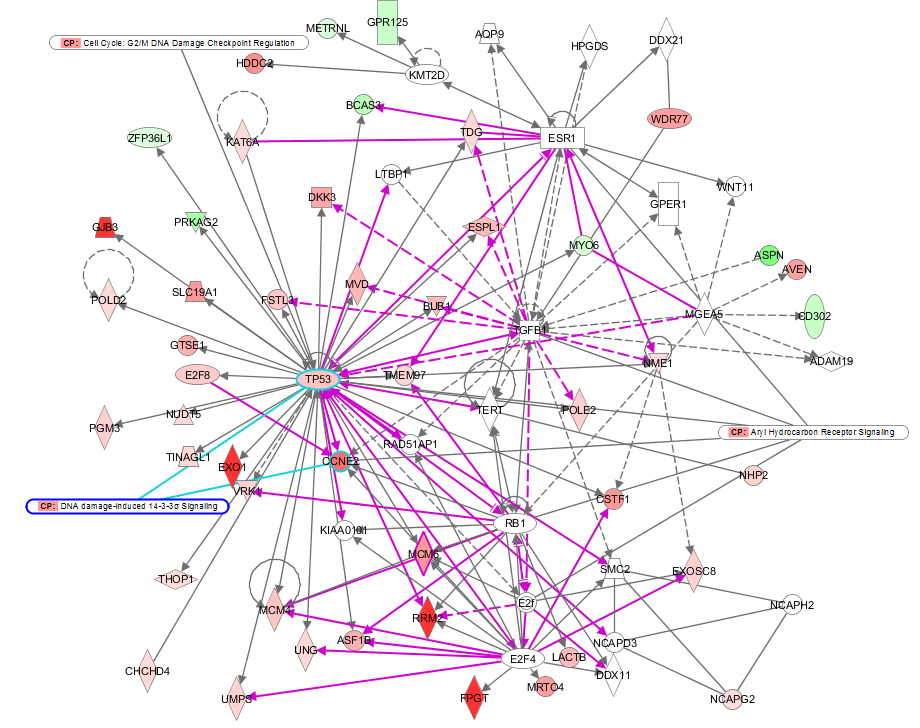


15h


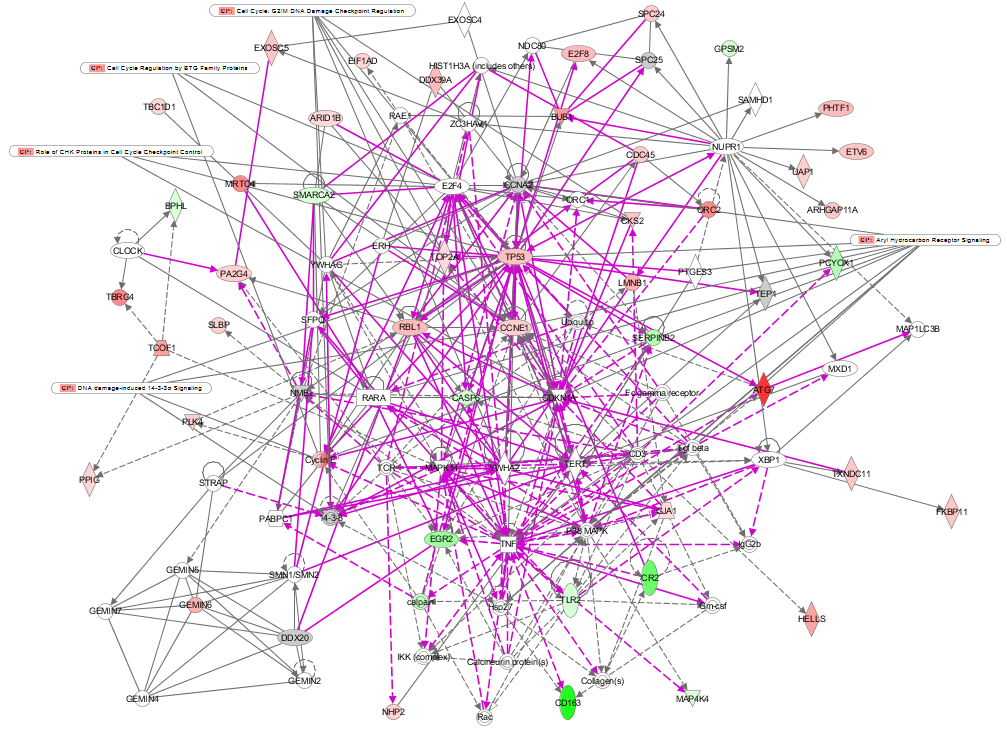


18h


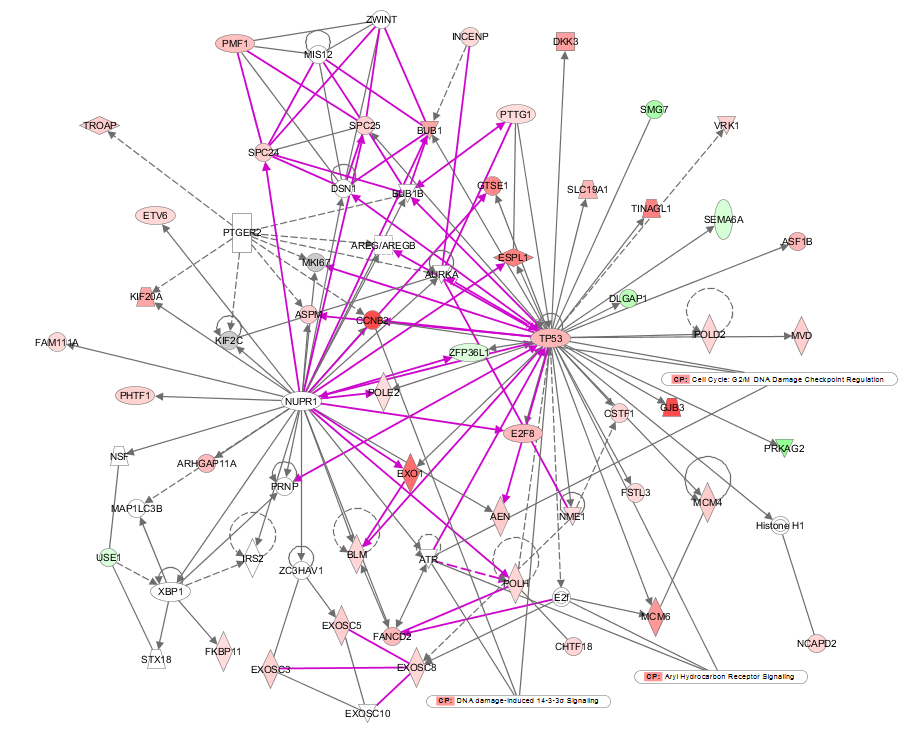


21h


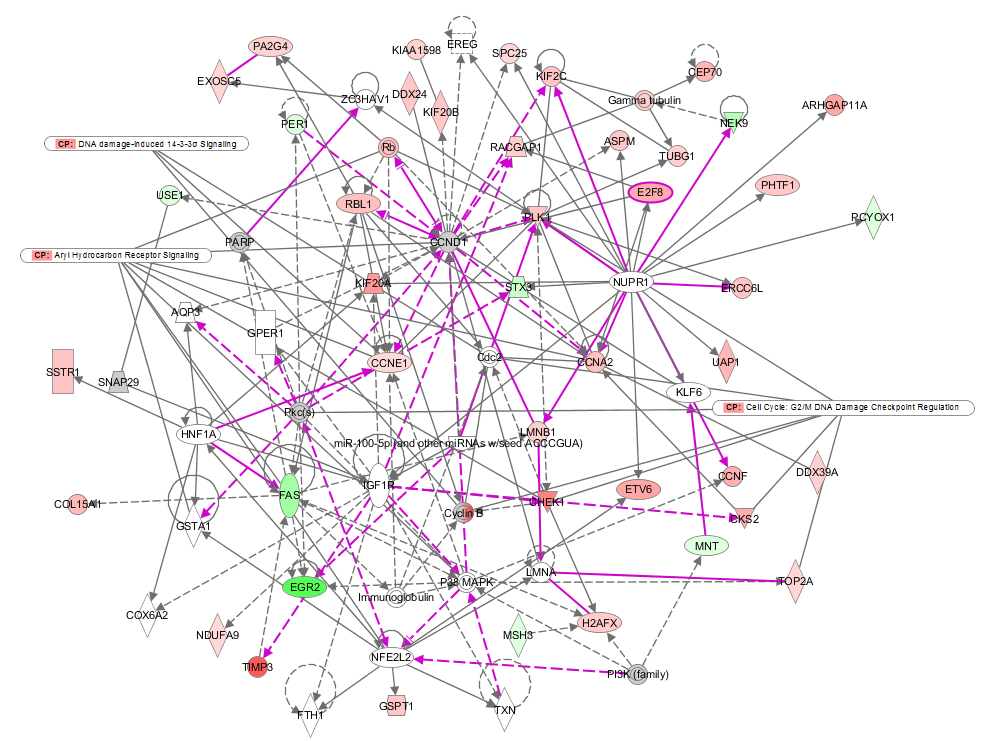


21.5h


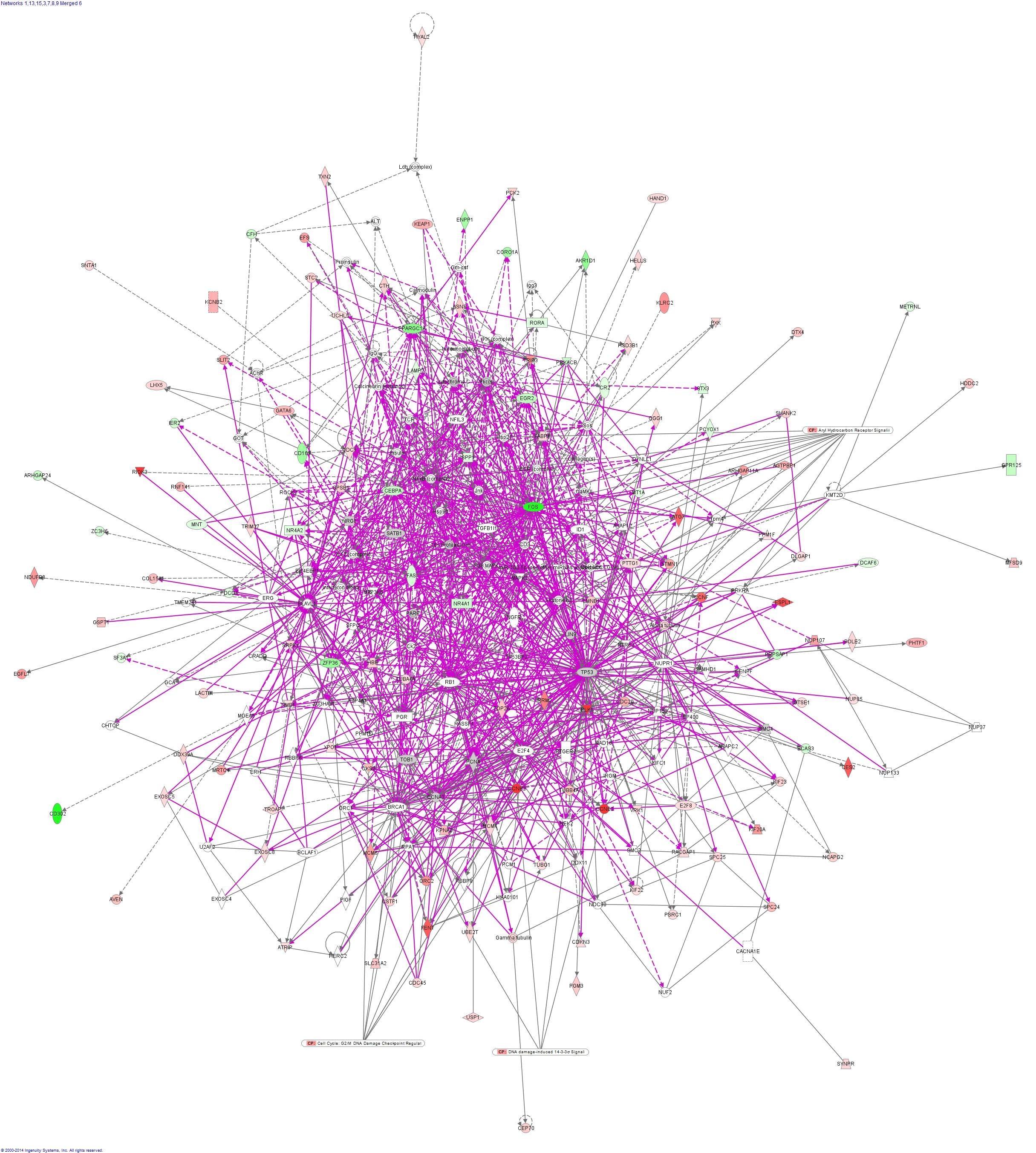


22h


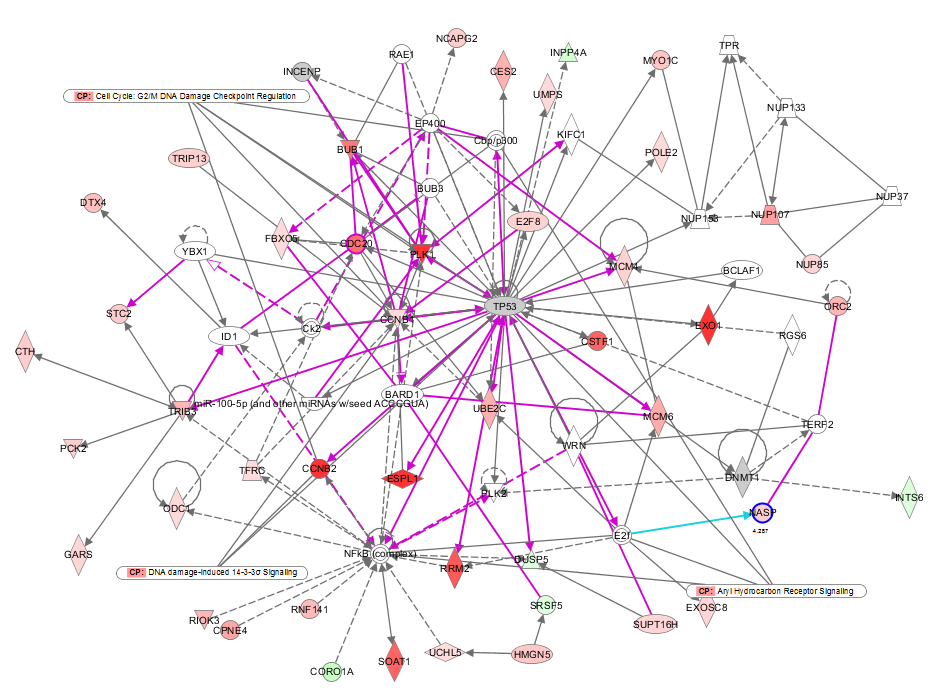


23.5h


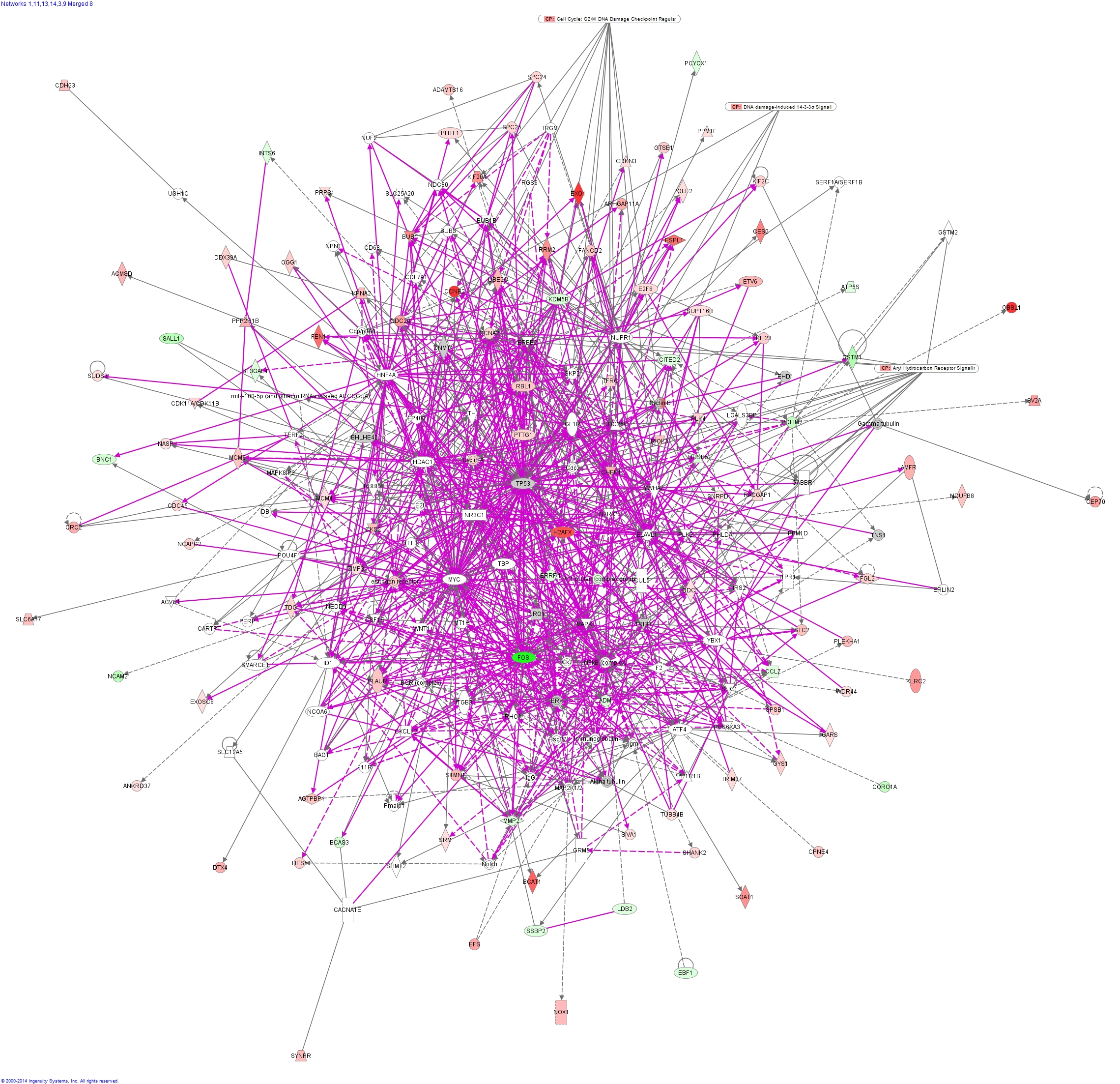


25h

Supplement: Additional file 4: Figure S3. — The interaction network between signaling pathway and cell cycle network. (DOC 9794 kb) [file 12860_2015_71_MOESM4_ESM.doc]
