# Supplementary material for: Analysis of the ways and methods of signaling pathways in regulating cell cycle of NIH3T3 at transcriptional level
Source: BMC Cell Biol. 2015 Oct 28;16:25. doi: 10.1186/s12860-015-0071-7 (PMC4625951; doi:10.1186/s12860-015-0071-7)
Supplement: Additional file 3: Figure S2. — Signal transduction of the signaling pathway that the reported genes involved (DOC 4343 kb) [file 12860_2015_71_MOESM3_ESM.doc]

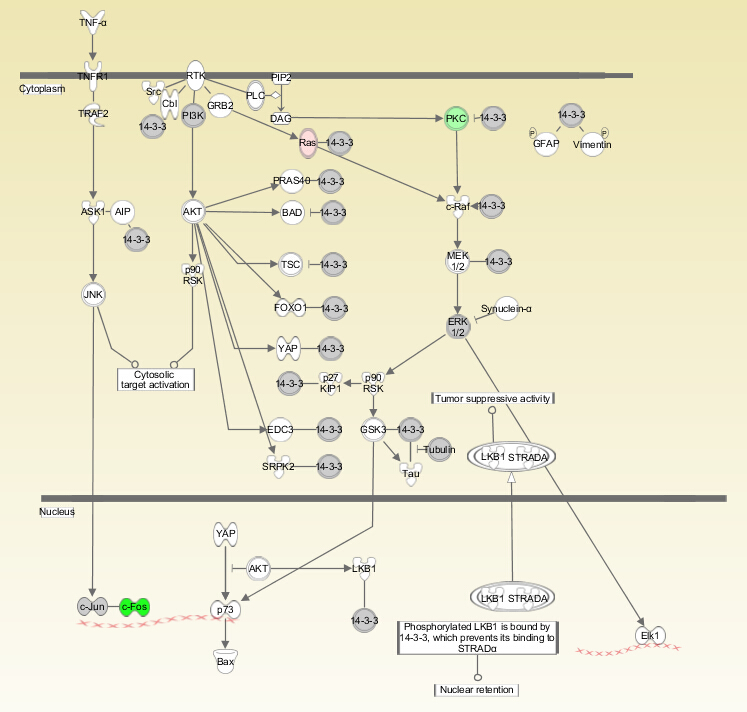


14-3-3-mediated Signaling (15h)


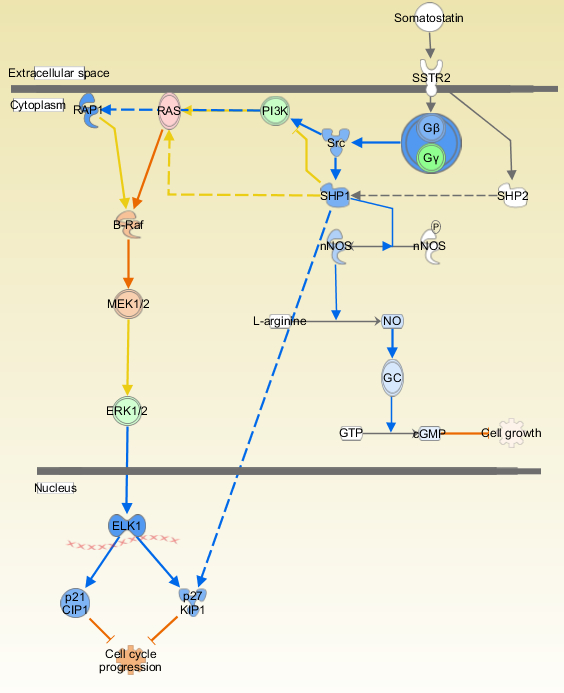


Antiproliferative Role of Somatostatin Receptor2(10h)


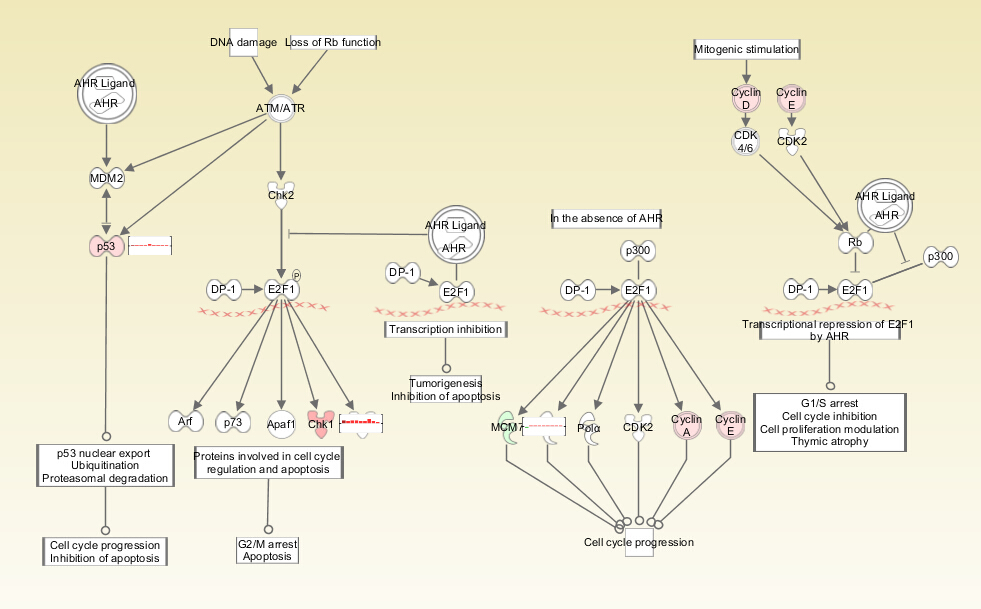


Aryl Hydrocarbon Receptor Signaling


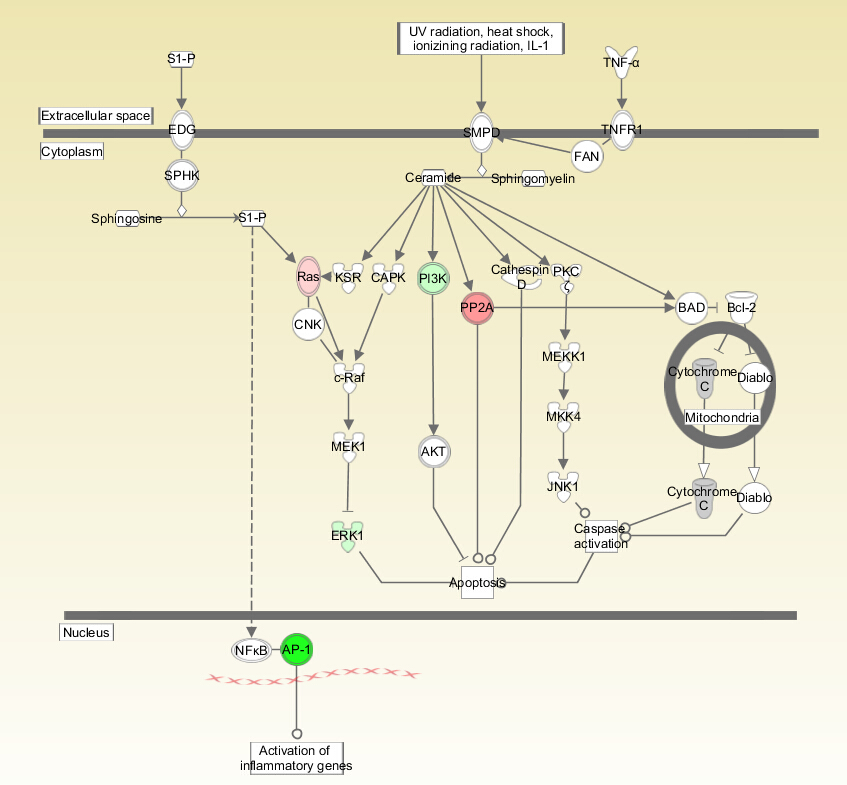


Ceramide Signaling (10h)


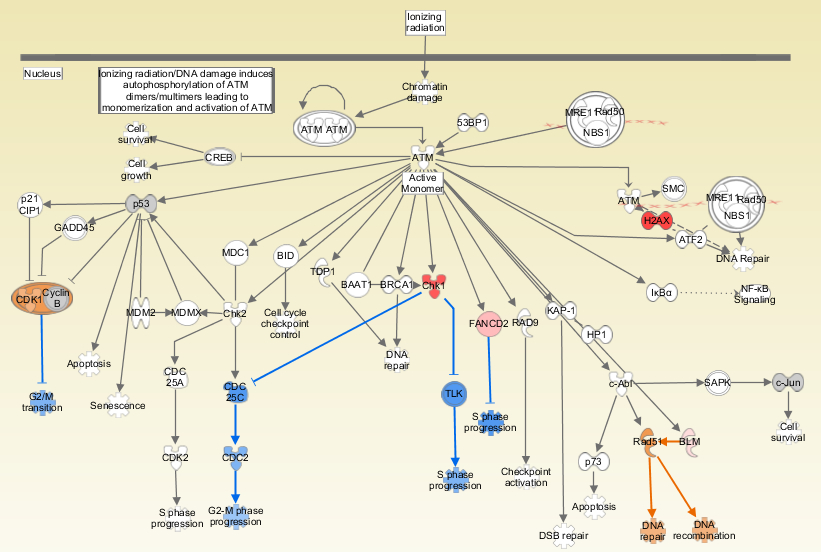


ATM Signaling (10h)


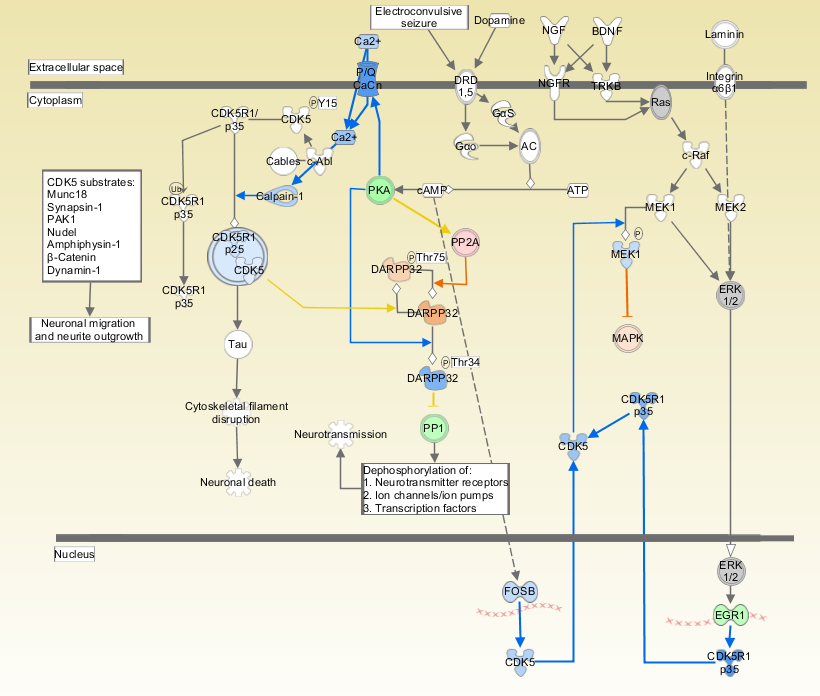


CDK5 Signaling (18h)

Cell Cycle Control of Chromosomal Replication (21.5h)


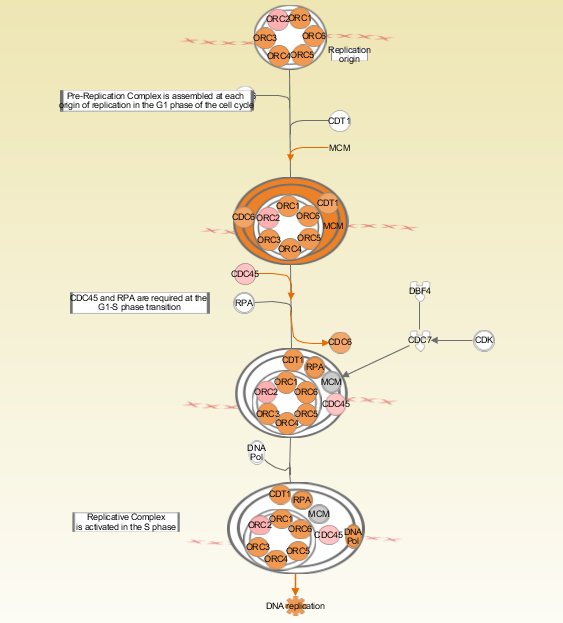


Cyclins and Cell Cycle Regulation (15h)


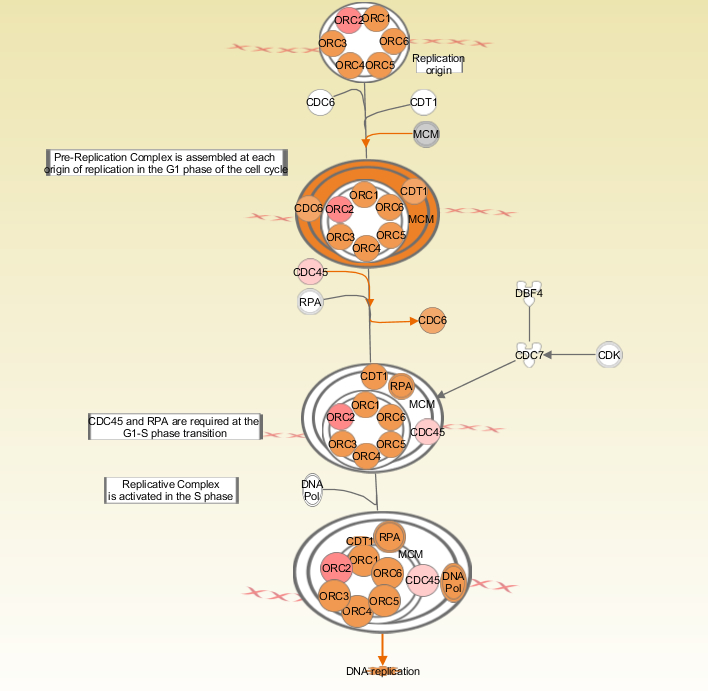

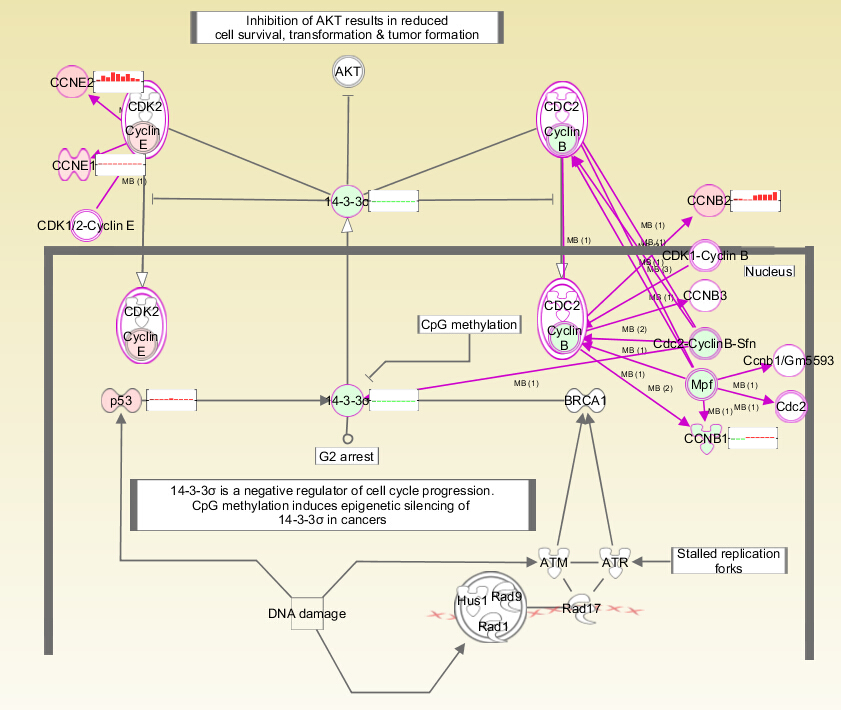


DNA damage-induced 14-3-3σ Signaling (22h)


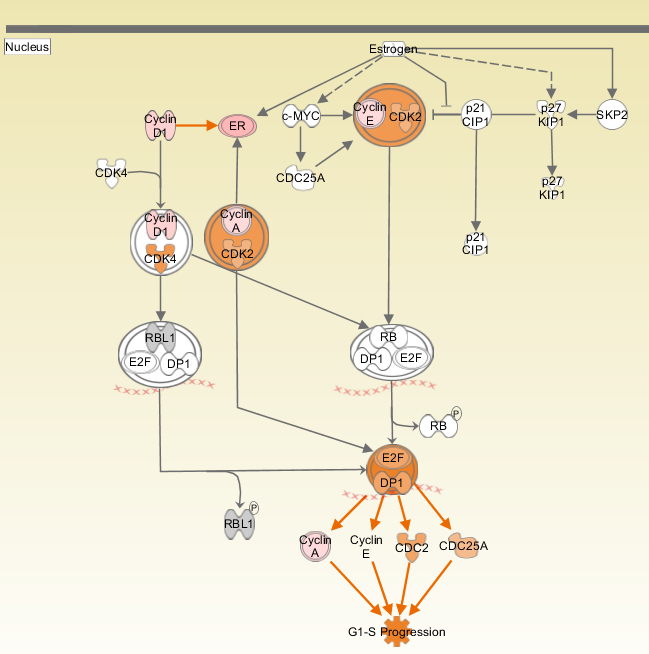


Estrogen-mediated S-phase Entry (15h)


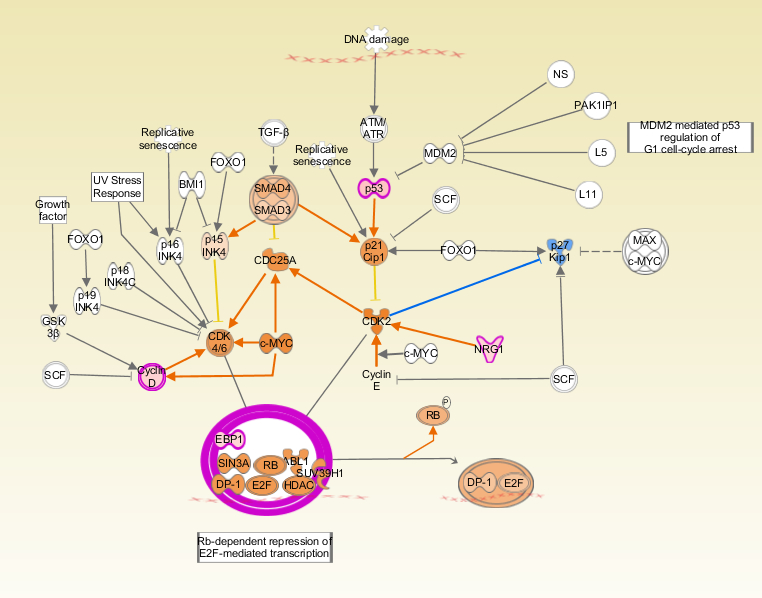


G1/S Check Point Regulation (15h)

G2/M DNA Damage Check Point Regulation (22h)


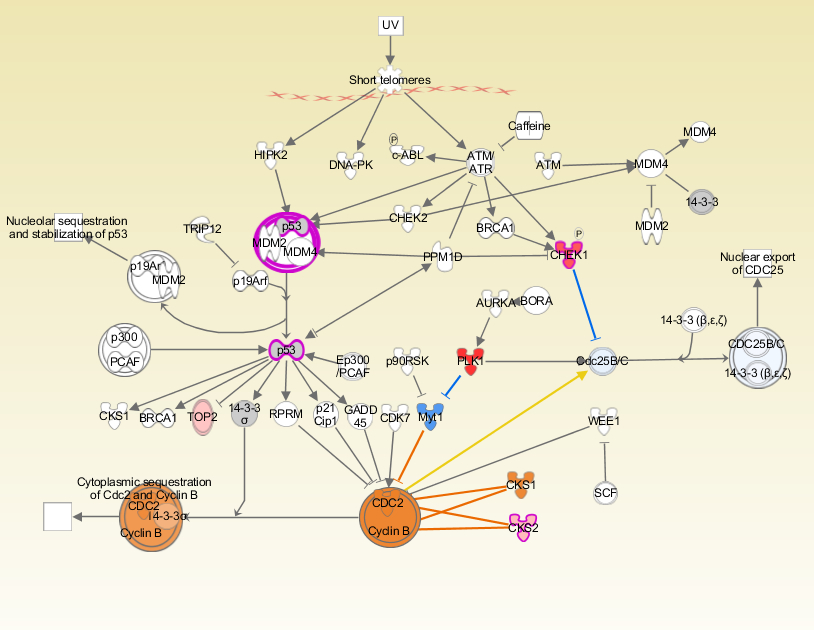

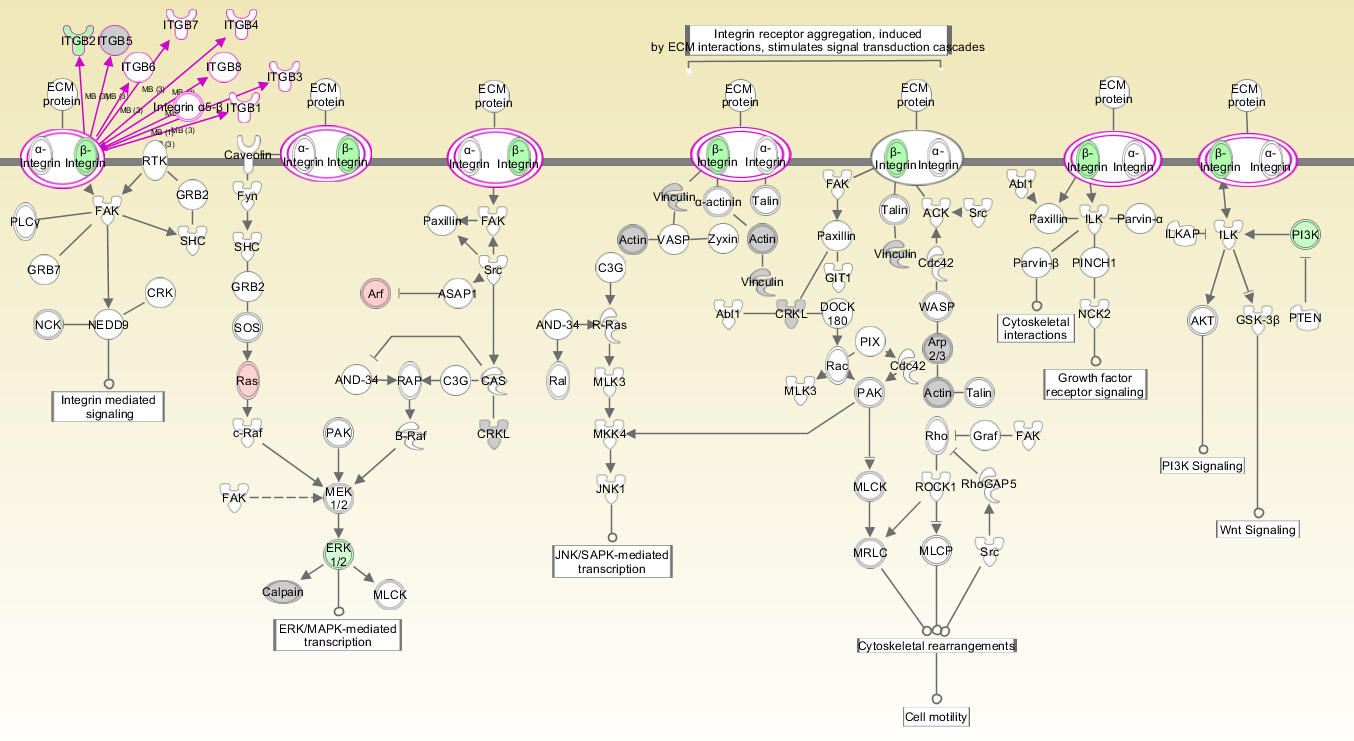


Integrin Signaling (18h)


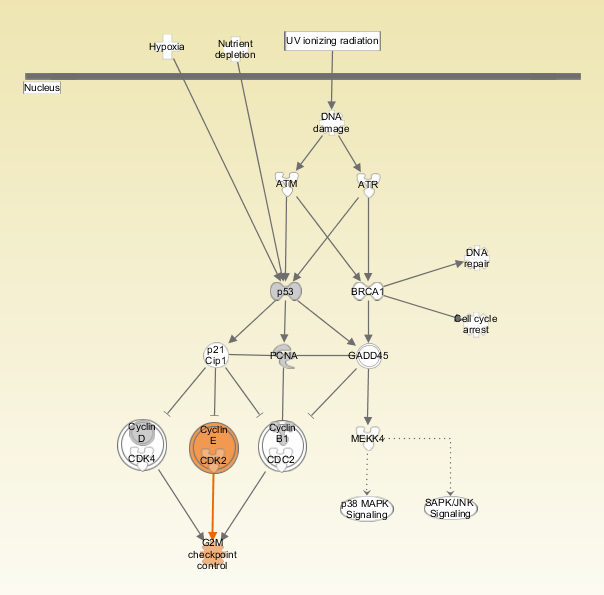

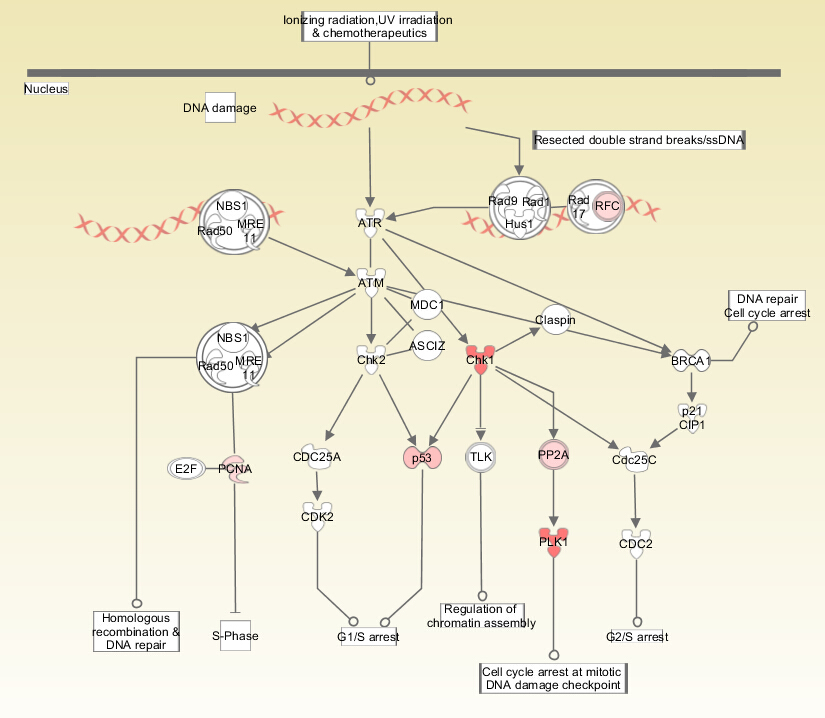


Role of CHK Proteins in Cell Cycle Checkpoint Control (18h)


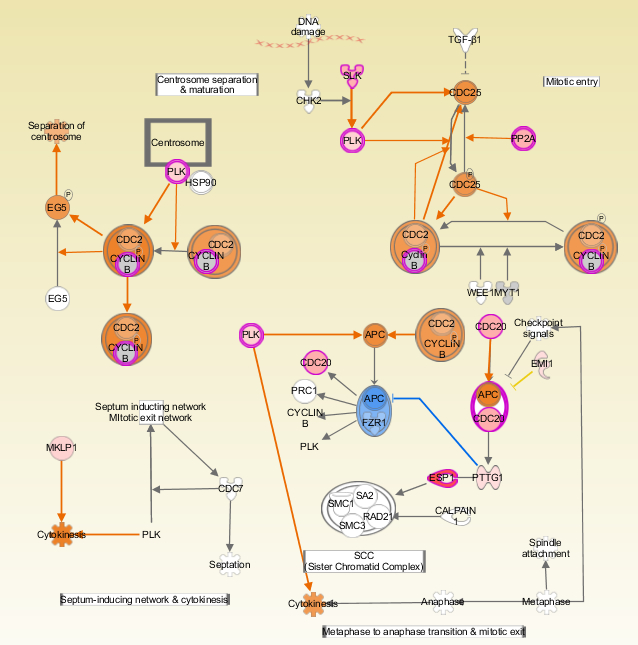


Mitotic Role of Polo-Like Kinase (22h)

GADD45 Signaling (22h)


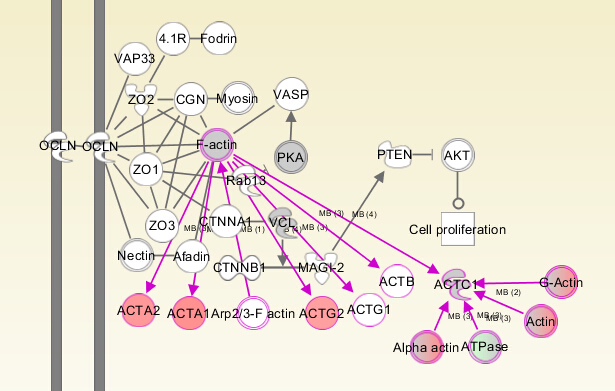


Tight Junction Signaling (22h)

**A** ，**E** Antiproliferative Role of Somatostatin Receptor2 ，**F** Aryl Hydrocarbon Receptor Signaling 在转录水平的表达变化，**I** CDK5 Signaling在蛋白水平的表达变化 (25h), **J** Ceramide Signaling 在转录水平的表达变化 (10h),  **K** Role of CHK Proteins in Cell Cycle Checkpoint Control 在转录水平的表达变化 (18h)，**M** DNA damage-induced 14-3-3σSignaling 在转录水平的表达变化, **N** DNA Methylation and Tra-scriptional Repression Signaling 在转录水平的表达变化 (22h), **O** G2/M DNA Damage Check Point Regulation在转录水平的表达变化, **P** Integrin Signaling 在转录水平的表达变化 (10h), **Q** Integrin Signaling 在转录水平的表达变化 (10h), **S** Tight Junction Signaling 在转录水平的表达变化 (10h),
